# Atypical Presentation of Gout: A Case Report

**DOI:** 10.7759/cureus.36707

**Published:** 2023-03-26

**Authors:** Ameen Abdel-Khalek, Ammarah Tariq, Joseph A White, Amara R Cheema, Sirjana Dhillon, Frederick Tiesenga

**Affiliations:** 1 Medicine, Saint James School of Medicine, Park Ridge, USA; 2 Internal Medicine, Ross University School of Medicine, Pembroke Pines, USA; 3 Medicine, Caribbean Medical University School of Medicine, Willemstad, CUW; 4 General Surgery, West Suburban Medical Center, Chicago, USA

**Keywords:** tophi, skin lesion biopsy, erythema nodosum, pseudogout, gout

## Abstract

Gout is the most common inflammatory arthritis which is caused by the buildup of uric acid crystals in the joints, that leads to severe pain, swelling, and stiffness. The condition typically affects the first metatarsophalangeal joint but it can impact other joints in the body. We present a case in which a 43-year-old male with a past medical history of obesity, hypertension, osteoarthritis, and gout presented with bilateral leg pain and the inability to walk for the last two years. Labs showed persistent leukocytosis, elevated ESR (erythrocyte sedimentation rate), normal uric acid levels, with physical exam findings of bilateral tender nodular leg lesions. Chest X-ray, head CT without contrast, left hip X-ray and ultrasound of left lower extremity were performed which were all negative. Biopsy of the tender skin nodules confirmed the diagnosis of tophaceous gout. Acute and prophylactic treatment of tophaceous gout resulted in resolved inflammation and leukocytosis without any complications.

## Introduction

Physicians worldwide have ample research referencing risk factors contributing to gout, thus it is no surprise to again suggest the common risk factors. However, what makes gout interesting, is the overlap of numerous differential diagnoses that may hide the underlying diagnosis of gout. In fact, some studies call it the great mimicker. Although high levels of uric acid is the biochemical fault, there are many patients who don’t even form crystals. In fact, 5% of those who develop gout, have uric acid levels above 9 mg/dL [1]. In a study done in 2009, 339 patients who presented with acute gout, 14% had serum uric acid levels ≤6 mg/dL, and 18% had levels between 6 mg/dL and 8 mg/dL [2]. The later presentation usually involves nephrolithiasis and tophi, as well as recurrent episodes of acute inflammatory attacks affecting the quality of life. However, it is still one of the most ubiquitous diseases on earth. To further provide evidence of the pervasiveness of gout, as part of the GBD 2010 Study, the Musculoskeletal Disorders and Risk Factors Expert Group estimated gout to be a global burden [3]. Essentially, this burden of gout is further perplexed by the atypical presentations that are most commonly present in patients aged >65 years old with comorbid conditions [4]. Although there is a rising public health burden, suboptimal management leads to increased morbidity, along with, “substantial healthcare utilization and cost burden” [3].

Monosodium urate (MSU) crystal deposition disease presents with extracellularly deposits, most commonly in the joints, which is a well-known type of arthritis treated with NSAIDs (non-steroidal anti-inflammatory drugs) and Colchicine in the early stages [1]. In fact, modern management involves a cocktail of regimens to assist in prevention, dissolution, and target therapy. Lifestyle modifications are discussed with patients to improve the quality of life.

Here we discuss the case of a 43-year-old male with a past medical history of arthritis, obesity, gout, hypertension, and suggestive chronic inflammation due to the persistent leukocytosis, elevated erythrocyte sedimentation rate (ESR), and bilateral tender nodular leg lesions. Occupational/educational history was not revealed by the patient. Uric acid levels were determined to be normal in the patient hence prompting several differential diagnoses. Sarcoidosis and its subcutaneous complication, erythema nodosum, along with pseudogout are important considerations which can contribute to the patient's presentation and pathogenesis.

## Case presentation

Chief complaint

Our patient is a 43-year-old male reporting bilateral leg pain and inability to walk for several years. Fatigue and tenderness in the nodules along the legs bilaterally has increased dramatically throughout the week which prompted the patient to present to the Emergency Room (ER).

History of present illness

A 43-year-old male presented to the Emergency Room with complaints of bilateral leg pain and inability to walk. The patient stated that he has not been able to walk for two years due to pain and has used crutches daily. The patient has tenderness present in the knee and ankle with no back or hip pain. The patient states that he has never smoked in the past and does not consume alcohol. He also reported several nodular tender lesions on his legs which have become bothersome. The patient admits that he is noncompliant with medications, yet he has never had to come to the ER since his "pain episodes eventually subside". Even though the patient stated increased fatigue and tenderness along the legs he denies any fever or chills. The patient is admitted for inability to self-care. Infectious disease, orthopedic surgery, and social workers were consulted due to his chronic conditions.

Past medical history

The patient’s past medical history includes obesity, arthritis, gout and hypertension. His chronic arthritis and gout were previously managed by NSAIDs and Allopurinol yet the patient is not adherent. Although these chronic conditions are present, the patient at no time had to come to the Emergency Room. The patient cannot recall the last time he was examined by a medical professional. Past surgical history consists of left quadricep tendon repair with no complications.

Examinations

On examination, the patient was found to have 2+ bilateral edema of the lower extremities. Bilateral nodular lesions with induration were present along with mild tenderness with palpation of the left knee and ankle. Discomfort is more prominent with left leg movement along with elevated trace left knee effusion compared to the right. Skin color, turgor, and texture are normal. The patient is very deconditioned. He is unable to ambulate without the assistance of crutches.

Preliminary differentials

Preliminary differentials were gout, sarcoidosis, erythema nodosum, and pseudogout. Gout was the initial clinical diagnosis due to the patient's obvious previous medical history and medication noncompliance. Yet normal uric acid levels prompted further investigation.

Investigations

Infectious disease was consulted due to the patient’s labs which demonstrated persistent leukocytosis with neutrophil predominance without any fever or chills. The etiology of leukocytosis and nodular skin lesions is still unclear. Blood cultures were obtained and determined to be inconclusive. Hypocalcemia, hyperphosphatemia, elevated ESR, elevated procalcitonin, and normal uric acid are shown in the patient's labs. Chest X-ray, head CT without contrast, left hip X-ray and ultrasound of left lower extremity were all negative. A biopsy of the tender skin nodules was done in order to determine the etiology.

Outcome/Progression

An abnormal skin lesion over the left leg was excised in elliptical fashion approximating 4 x 1 cm including skin and subcutaneous full thickness. The specimen was sent for pathology which confirmed the clinical diagnosis of tophaceous gout. The patient continued to receive inpatient treatment to insure medical compliance and resolution. Other chronic medical conditions were also addressed regarding orthopedics and rheumatology. Leukocytosis and inflammation resolved with acute and prophylactic treatment of gout.

Treatment

The patient was treated with Allopurinol 200 mg po (orally) daily and Indomethacin 50 mg po bid (twice a day) with food. Colchicine 0.6 mg po q2hr (every two hours) prn (as needed). The patient was counseled on a medication regimen along with lifestyle modifications in order to improve the quality of life.

## Discussion

Gout is an inflammatory reaction due to the accumulation of monosodium urate crystals in various joints but most commonly presents on the first metatarsophalangeal joint. An article reviewed in Nature Reviews Rheumatology in 2020 published the prevalence of gout ranged from <1% to 6.8% with an incidence of 0.58-2.89 per 1,000 person-years [2]. This number varies depending on the population that is being studied. The burden of gout has increased globally in the past 50 years [2]. The Global Burden of Disease Study 2017 published that there are 41 million living with gout worldwide [3]. The prevalence of gout in 1990 was 20.2 million, and this number more than doubled in 2017 to 41.2 million. Even with the advances in the treatment of gout, a failure to comply with a treatment regimen has led to these increasing numbers [5].

There are many risk factors which can lead to gout. Genetic mutations in the renal urate transporter system may be correlated with under-excretion or overproduction of uric acid crystals which can lead to gout nodules known as tophi [1]. Other risk factors include increased age, along with a family or personal history of gout attacks, osteoarthritis, consumption of alcohol, purine-rich foods and medications such as thiazide diuretics for hypertension may precipitate gout in highly susceptible individuals [3]. Thiazide and thiazide-like diuretics were associated with an increased risk of incident gout caused by the surge in serum uric acid concentration which was prevalent a few days after the initiation of therapy, the clinical features of gout and drug-induced gout do not differ [3]. The patient being presented discontinued any hypertension medication during his inpatient management, thus excluding diuretics as a possibility and secondary causation of gout; Serum uric acid levels were normal in the patient making this an unlikely diagnosis.

Nodules presenting as periarticular masses are usually uncommon and encourage investigation. A differential diagnosis should be taken into consideration. Erythema nodosum presents as a superficial inflammatory infiltrate predominantly composed of lymphocytes seen on the dermis leading to the term septal panniculitis [6]. A common differential for crystalline tophus should include tumoral calcinosis and tophaceous pseudogout, which both consist of soft tissue calcification hence making them radiopaque [7]. A definitive diagnosis of pseudogout can be confirmed by identifying CPPD (calcium pyrophosphate deposition) crystals in the synovial fluid which are intensely basophilic and rhomboid-shaped [8]. Tumoral calcinosis is amorphous compared to gout and pseudogout; an important distinguishing feature is that gout exhibits monosodium urate crystals which leads to a strong negative birefringence compared to the weakly positive birefringence in pseudogout [9].

In the case of erythema nodosum or any abnormal nodule an initial blood count, sedimentation rate, antistreptolysin titers, urinalysis, throat culture, and chest roentgenogram should be obtained to help distinguish and confirm a clinical diagnosis [8]. Nonspecific symptoms such as persistent leukocytosis, elevated ESR, and multiple skin lesions can make it difficult to differentiate gout from other possible diagnoses, hence a biopsy of a skin lesion or synovial fluid aspiration (which is the gold standard) may be indicated in order to confirm the suspicion of gout [10].

Only about 46% of adults diagnosed with chronic gout are adherent to their medication [3-11]. It is important to mention that the patient lives in a homeless shelter and has an unhealthy lifestyle which can contribute to his medication noncompliance. Including the patient's history, this can lead to long-term complications. These complications include erosion, uric acid nephrolithiasis, and destruction of a joint along with deposits of urate crystals under the skin (tophi). Tophi manifest as tender nodules when an individual is suffering from a gout attack, which is due to the rise in uric acid levels, whereas the patient being presented did not exhibit any signs of a traditional gout attack (normal uric acid levels). Hence the tender nodules and leukocytosis accompanied by the patient required further analysis.

## Conclusions

The patient sustained normal serum uric acid levels thereupon leading to differential diagnoses other than gout. A negative chest X-ray and hypocalcemia on the patient’s labs prompt a diagnosis other than sarcoidosis and its subcutaneous complication erythema nodosum. Although the patient had nonspecific symptoms such as persistent leukocytosis and tender skin nodules, pseudogout was excluded due to the skin lesion biopsy which determined the clinical diagnosis, tophaceous gout.

Management of the patient was based on the nodular skin lesion biopsy findings and the patient's history leading to acute and prophylactic treatment of tophaceous gout. Despite the fact that the patient has other chronic medical conditions, he was treated accordingly with minimal to no complications. Leukocytosis and inflammation resolved with acute and prophylactic therapy of gout.
